# Supplementary material for: Splice-Junction-Based Mapping of Alternative Isoforms in the Human Proteome
Source: Cell Rep. Author manuscript; Available in PMC 2020 Jan 15. (PMC6961840; doi:10.1016/j.celrep.2019.11.026)
Supplement: 2 [file NIHMS1546469-supplement-2.zip › DF1/PXD010154/Testis/Testis_54_DDAH2_WHRCSHALIRGVPESLASGEGAGAGLPALDLAK.pdf]

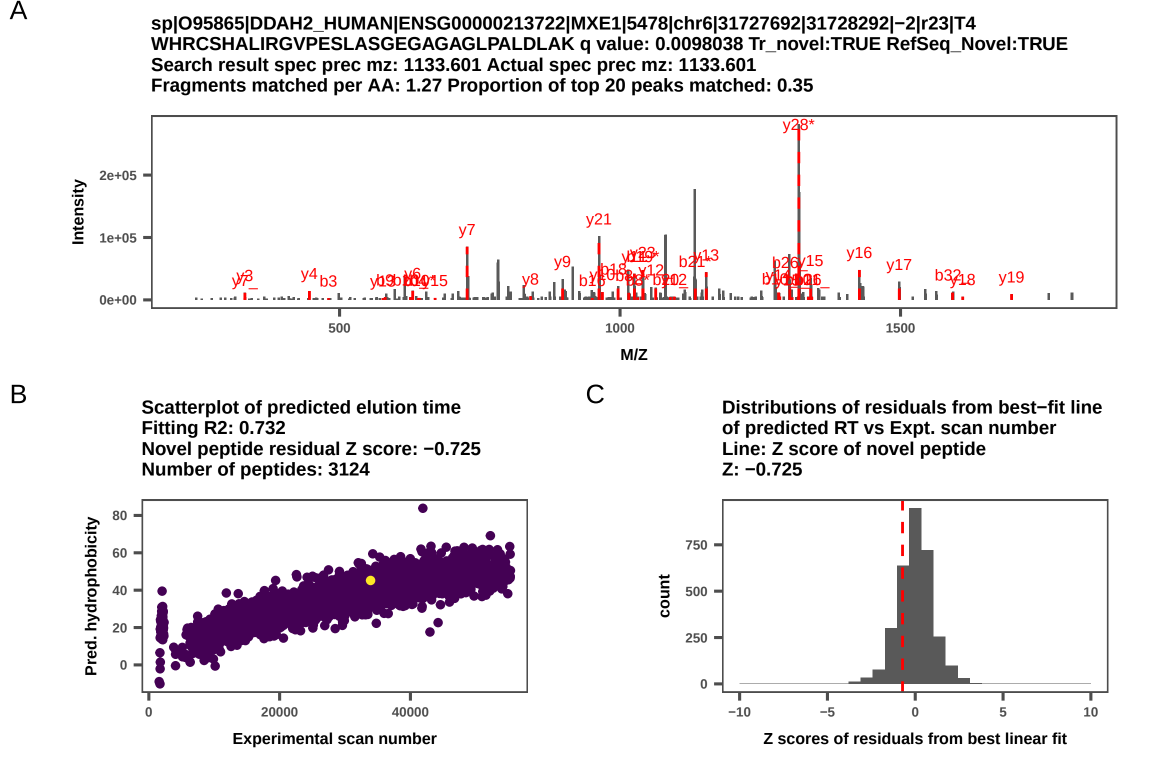
A
sp|O95865|DDAH2_HUMAN|ENSG00000213722|MXE1|5478|chr6|31727692|31728292|−2|r23|T4
WHRCSHALIRGVPESLASGEGAGAGLPALDLAK q value: 0.0098038 Tr_novel:TRUE RefSeq_Novel:TRUE
Search result spec prec mz: 1133.601 Actual spec prec mz: 1133.601
Fragments matched per AA: 1.27 Proportion of top 20 peaks matched: 0.35
Intensity
2e+05
1e+05
0e+00
500
1000
1500
M/Z
y28*
y21
y7
y16
y17
y19
y9
y8
y4
b3
y3
b32
y18
b26
y15
y13
y12
B
Scatterplot of predicted elution time
Fitting R2: 0.732
Novel peptide residual Z score: −0.725
Number of peptides: 3124
Pred. hydrophobicity
80
60
40
20
0
0
20000
40000
Experimental scan number
C
Distributions of residuals from best−fit line
of predicted RT vs Expt. scan number
Line: Z score of novel peptide
Z: −0.725
count
750
500
250
0
−10
−5
0
5
10
Z scores of residuals from best linear fit
